# Study on the effects of estimated dose of radiation to immune cells and CBC parameters on the prognosis of patients with locally advanced non-small cell lung cancer undergoing radiotherapy

**DOI:** 10.3389/fimmu.2026.1852238

**Published:** 2026-08-19

**Authors:** Aihui Feng, Wuyan Xia, Yan Shao, Hao Wang, Hua Chen, Hengle Gu, Yanhua Duan, Ying Huang, Zhenjiong Shen, Jiaou Chen, Zhiyong Xu

**Affiliations:** Shanghai Chest Hospital , Shanghai Jiao Tong University School of Medicine, Shanghai, China

**Keywords:** CBC parameters, EDRIC, LA-NSCLC, PLR, survival outcomes

## Abstract

**Background:**

Locally advanced non-small-cell lung cancer (LA-NSCLC) is a subtype of lung cancer with substantial heterogeneity in clinical prognosis. Immune injury induced by radiotherapy exposure is a critical factor affecting treatment response and long-term outcomes. This study enrolled patients with LA-NSCLC to investigate the prognostic value of complete blood count (CBC) parameters combined with the estimated dose of radiation to immune cells (EDRIC), aiming to facilitate precision prognostic stratification and tailored treatment strategies in clinical practice.

**Materials and methods:**

This study included 329 patients with LA-NSCLC who received radiotherapy (RT) at our institution. Serial CBC measurements were obtained pre-RT, weekly during RT, and 1-month post-RT. EDRIC was calculated based on the model developed by Jin et al. and improved by Ladbury et al. We subsequently evaluated the associations of EDRIC and CBC parameters with overall survival (OS), local recurrence-free survival (LRFS), distant metastasis-free survival (DMFS), and progression-free survival (PFS).

**Results:**

Univariate and multivariate analyses demonstrated that EDRIC, together with week 3 platelet-to-lymphocyte ratio (PLR), was significantly associated with OS, LRFS, DMFS, and PFS (*P* < 0.05). In multivariate models, neither MHD, MLD, nor MBD was significantly associated with survival outcomes, regardless of the inclusion of EDRIC; however, EDRIC demonstrated a significant correlation. For OS discrimination, Harrell’s C-index reached 0.622 for the EDRIC-alone model, 0.734 for the model integrating clinical covariates and CBC markers, and 0.740 for the combined model incorporating clinical features, serial CBC indices and EDRIC.

**Conclusions:**

EDRIC is an independent prognostic biomarker for OS, LRFS, PFS and DMFS in LA-NSCLC patients undergoing definitive thoracic (chemo)radiotherapy. Week 3 PLR also independently predicts all four survival endpoints. Compared with the EDRIC-only model, the integrated model incorporating pretreatment EDRIC, clinical variables, and on-treatment inflammatory markers (NLR and PLR) yields more accurate and comprehensive prognostic stratification for patients with LA-NSCLC.

## Introduction

The incidence and mortality rates of lung cancer rank first among malignant tumors in China (1). Non-small-cell lung cancer (NSCLC) accounts for approximately 85% of lung cancer cases, with most patients diagnosed at advanced stages and exhibiting a low 5-year survival rate (2). Unresectable advanced NSCLC is a highly heterogeneous and complex group of cancers, representing approximately 30% to 40% of newly diagnosed cases. The 5-year overall survival (OS) rate following concurrent chemoradiotherapy (cCRT) or sequential chemoradiotherapy (sCRT) remains around 20% (3). Notably, the real-world PACIFIC-R study published as 5-year outcomes with durvalumab after chemoradiotherapy in unresectable stage III NSCLC delivered encouraging long-term survival data by adding durvalumab consolidation following chemoradiotherapy. With a median OS follow-up of 67.5 months, this large cohort reported a markedly improved 5-year OS rate of 49.2% (4).

The remarkable survival benefit derived from integrating immunotherapy after radiotherapy has prompted extensive mechanistic research on chemoradiotherapy-immunotherapy combinations. Cumulative evidence reveals that radiotherapy (RT) can reshape the tumor immune microenvironment (5), trigger endogenous anti-tumor immune responses (6), and promote tumor elimination, thereby synergistically enhancing the anti-cancer efficacy of immune checkpoint inhibitors (ICIs). Beneficial distant effects, known as the abscopal effect, have been observed in both animal (7, 8) and clinical (9) studies, characterized by the shrinkage of unirradiated tumors outside the radiation field. However, RT can also exert immunosuppressive effects, one of the most clinically significant being radiation-induced lymphopenia (10). Cho et al. reported, based on clinical outcomes of 73 patients with limited-stage small-cell lung cancer, that radiation-related lymphopenia is associated with poor survival (11). Pike et al. (12) analyzed prognostic data from 225 lung cancer patients receiving palliative RT across five institutions and demonstrated that lymphopenia was associated with inferior survival in patients treated with ICIs. Compromised immune function may attenuate the therapeutic efficacy of chemoradiotherapy, which in turn results in poorer survival outcomes. Consequently, the role of the immune system in RT has garnered increasing attention, and the immune system may represent a potential organ at risk (OAR) warranting protection during treatment.

In previous RT practices, the immune system was not considered a significant OAR. However, a growing number of recent studies (13, 14) have demonstrated that radiation-induced immune toxicity may play a crucial role in tumor progression. To address this, Jin et al. proposed the concept of the estimated dose of radiation to immune cells (EDRIC) (15), which incorporates the mean lung dose (MLD), mean heart dose (MHD), mean body dose (MBD), beam-on time, and radiation fractionation. In the prognostic evaluation of lung cancer radiotherapy, EDRIC can be used to assess the potential risk of damage from thoracic RT to systemic and local immune organs, such as mediastinal lymph nodes and bone marrow. It is an important factor influencing both the OS and progression-free survival (PFS) rate of patients undergoing RT (16, 17). However, the EDRIC framework in its current form fails to capture the dynamic alterations in immune function following RT, particularly lacking the capacity to directly quantify radiation-induced immune injuries such as lymphopenia and bone marrow hematopoietic suppression—changes that bear profound implications for recurrence risk and long-term quality of life among patients with lung cancer.

Complete blood count (CBC) parameters, can directly reflect the immune cell function and hematopoietic status of lung cancer patients. A sustained decrease in lymphocytes during RT indicates suppression of the patient’s immune function, which may increase the risk of tumor progression (18). Therefore, the integration of EDRIC and CBC parameters enables the evaluation of the potential long-term impact of irradiation on immune function, as well as the real-time monitoring of the patient’s immune and hematopoietic status during RT. Ultimately, this approach will enable the development of a more accurate and comprehensive prognostic model for lung cancer patients, providing a basis for individual treatment adjustments, such as optimizing radiotherapy plans and guiding immunotherapy interventions.

In this retrospective study, we conducted a comprehensive survival analysis on an independent cohort of LA-NSCLC patients who received RT at our institution, aiming to validate the independent prognostic value of EDRIC and further clarify its clinical significance. Building on this, we developed a new dynamic lung cancer prognosis prediction model by integrating EDRIC with dynamic CBC parameters monitoring throughout RT. We anticipate that the model proposed in this study will surpass EDRIC in predictive accuracy and provide valuable data for individualized prognostic stratification and treatment decision-making in LA-NSCLC patients.

## Materials and methods

### Patients

This retrospective analysis included patients with LA-NSCLC who received definitive RT with or without chemotherapy at Shanghai Chest Hospital between 2012 and 2019. The inclusion criteria were as follows: (1) pathologic confirmation of NSCLC with clinical stage III disease according to the TNM criteria of NSCLC (Union for International Cancer Control, eighth edition); (2) receipt of chest RT with a dose of at least 50 Gy delivered in once-daily fractionated sessions; (3) availability of complete RT data; and (4) availability of baseline pretreatment CBC data. Serial CBC measurements before RT, weekly during RT, and at 1 month after radiotherapy were collected for dynamic analysis but were not mandatory for patient enrollment. Patients were excluded if they: (1) lacked complete radiotherapy dose parameters or baseline CBC data; (2) received systemic corticosteroid therapy at the time of CBC acquisition; or (3) had a history of autoimmune diseases. In total, 418 patients with LA-NSCLC receiving definitive radiotherapy were initially screened. After applying the predefined inclusion and exclusion criteria, 329 patients were finally included in the present analysis.

### Data collection

Data on CBC parameters and baseline clinical features were retrospectively extracted from electronic medical records (EMRs). Routine hematological indices comprised the neutrophil-to-lymphocyte ratio (NLR) and platelet-to-lymphocyte ratio (PLR). Baseline clinical covariates included age, gender, Eastern Cooperative Oncology Group performance status (ECOG PS), Charlson comorbidity index (CCI), smoking history, body weight loss, tumor histology, TNM stage, gross tumor volume (GTV), treatment sequence (radiotherapy alone, sequential chemoradiotherapy, and concurrent chemoradiotherapy), and chemotherapy regimen (no chemotherapy, pemetrexed-containing regimens, and non-pemetrexed chemotherapy regimens), the total number of chemotherapy cycles, and other clinical variables.

Radiotherapy parameters, including radiation techniques (3D conformal radiotherapy [3DCRT] and intensity-modulated radiotherapy [IMRT]), as well as MLD, MHD and MBD were derived from the Pinnacle^3™^ treatment planning system (TPS, v9.10, Philips Medical Systems, Cleveland) and subjected to statistical analysis. EDRIC was computed using the following formula. The EDRIC framework was originally proposed by Jin et al. (15) and subsequently modified by Ladbury et al. (19).


$$EDRIC=0.12\times MLD+0.08\times MHD+[0.45+0.35\times 0.85\times {\left(\frac{Number\;of\;fractions}{45}\right)}^{\frac{1}{2}}]\times MBD$$

### Follow-up

All patients were followed up retrospectively in accordance with standard clinical protocols for LA-NSCLC. Post-treatment surveillance was conducted at 3-month intervals within the first two years, semiannually from year 3 to year 5, and yearly thereafter. Standard follow-up assessments included physical examinations and contrast-enhanced CT of the chest and upper abdomen (covering primary thoracic tumors, liver and bilateral adrenal glands), abdominal ultrasonography, and other routine laboratory tests. Supplementary imaging, including brain magnetic resonance imaging (MRI), was arranged for patients with suggestive symptomatic manifestations. The cutoff date for complete follow-up data collection was September 12, 2025.

All procedures involving human participants in this study were conducted in accordance with the Declaration of Helsinki (2013 revision). The study protocol was approved by the Institutional Review Board of Shanghai Chest Hospital (No. KS21002). Written informed consent was waived due to the retrospective design of this study.

### Statistical analysis

The primary endpoint was OS, defined as the interval from the initiation of RT to death from any cause or the last follow-up. Secondary endpoints included PFS, local recurrence-free survival (LRFS), and distant metastasis-free survival (DMFS). PFS was defined as the time from RT initiation to disease progression, death, or last follow-up. LRFS was measured from the start of RT to death from any cause, local disease progression at the primary tumor site, or last follow-up. DMFS was defined as the interval from RT initiation to distant metastasis, death from any cause, or last follow-up.

First, baseline inter-variable correlations were assessed to mitigate multicollinearity before prognostic screening. Chi-squared tests (Fisher’s exact test for sparse cells) were used for categorical variables, and Spearman’s rank correlation was applied for continuous variables. Cramer’s V was calculated to quantify correlation strength between categorical covariates. Variable pairs with absolute Spearman’s r > 0.7 or Cramer’s V > 0.5 were deemed highly collinear; for each collinear pair, the covariate with weaker clinical relevance was excluded in advance.

Subsequently, univariate Cox proportional hazards regression was performed to screen candidate prognostic predictors for OS, PFS, LRFS, and DMFS separately. The Benjamini–Hochberg (BH) false discovery rate correction was applied to adjust P-values generated from univariate Cox analyses. All covariates retaining BH-corrected *P* < 0.05 in univariate Cox analysis were reserved for multivariate modeling.

Notably, EDRIC exhibited strong pairwise correlations with MLD, MBD, and MHD, which precluded simultaneous inclusion within a single multivariate model. To eliminate collinearity bias, two separate multivariate Cox models were constructed: Model 1 incorporated MLD, MBD and MHD without EDRIC, while Model 2 included EDRIC and excluded the three traditional dosimetric metrics.

Kaplan–Meier survival curves and log-rank tests were used to compare survival outcomes stratified by key biomarkers and dosimetric indicators. Maximally selected rank statistics were adopted to identify optimal cut-off values for continuous predictive markers across all four survival endpoints, stratifying patients into high- and low-risk subgroups for clinical risk stratification interpretation.

Harrell’s C-index was calculated to quantify model discriminative capacity, and stratified 10-fold cross-validation was conducted to evaluate the internal robustness of the two multivariate Cox models. Time-dependent calibration curves at 12, 24, 36 and 48 months were plotted to compare predicted risk scores against observed event rates, with Wilson-type 95% confidence intervals of observed event rates visualized as error bars. A time-dependent Hosmer–Lemeshow goodness-of-fit test was further used to assess model calibration; patients were evenly divided into five risk quantiles based on predicted risk scores, yielding a test degree of freedom equal to 3 (*df* = number of risk groups − 2 = 5 − 2 = 3).

All statistical analyses were performed using R software (version 4.5.0; R Core Team, 2025) (20), and all statistical tests were two-sided; a corrected *P* < 0.05 was regarded as statistically significant.

## Results

### Patients’ characteristics

Records for a total of 329 patients with LA-NSCLC received a total radiation dose of at least 50 Gy were identified for this study. The median duration of follow-up was 23.2 months. **Table 1** lists the clinical characteristics of the patients. In total, 93.62% of patients received chemotherapy, and the most common chemotherapy strategy was sCRT. The number of chemotherapy cycles ranged from 0 to 18, with a mean of 4 cycles. The mean EDRIC was 7.49 ± 1.95 Gy (range, 1.95-14.03 Gy). Descriptive statistics of serial NLR and PLR are provided in **Supplementary Table 1**.

**Table 1:** Patient characteristics.

Characteristics	N=329 [range]	%
Age (year)	61 [22-86]	
Gender		
Male	300	91.19
Female	29	8.81
ECOG PS		
0	26	7.90
1	300	91.19
2	3	0.91
CCI	2 [0-7]	
Smoking index (pack-years)		
<600	136	41.33
≥600	170	51.67
Unknown	23	7.0
Body weight loss (in 6 months)		
<5%	322	97.87
≥5%	7	2.13
Histology		
Squamous	197	59.88
Adenocarcinoma	105	31.91
NOS	27	8.21
Clinical T stage^a^		
T1	57	17.33
T2	120	36.47
T3	70	21.28
T4	82	24.92
Clinical N stage^a^		
N0	8	2.43
N1	14	4.26
N2	217	65.96
N3	90	27.35
Clinical stage^a^		
IIIA	133	40.43
IIIB	172	52.28
IIIC	24	7.29
Gross tumor volume (cc)	190.29 [9.76-707.31]	
No of RT fractions	28 [20-30]	
Prescribed dose (Gy)		
< 60 Gy	56	17.02
≥ 60 Gy	273	82.98
EDRIC (Gy)	7.49 [1.95-14.03]	
Treatment sequence		
RT alone	21	6.38
Sequential chemoradiotherapy	184	55.93
Concurrent chemoradiotherapy	124	37.69
Chemotherapy regimen		
No chemotherapy	21	6.38
Pemetrexed-containing regimens	78	23.71
Non-pemetrexed chemotherapy regimens	230	69.91
Number of chemotherapies		
<4 cycles	94	28.57
≥4 cycles	235	71.43

ECOG PS, eastern cooperative oncology group performance status; CCI, charlson comorbidity index; NOS, not otherwise specified; RT, radiotherapy; EDRIC, estimated dose of radiation to immune cells.

^a^ Cancer staging was done with the American Joint Committee on Cancer TNM staging system (8^th^ edition).

### Univariate analysis

**Table 2** presents univariate Cox regression results for four survival endpoints. For OS, elevated EDRIC, larger GTV, advanced T stage, advanced clinical stage, radiotherapy alone, lower radiotherapy dose, and 3DCRT were significantly linked to inferior OS. Treatment sequence also exerted a significant prognostic impact on OS. Serial hematological indices (NLR and PLR) obtained at baseline and weeks 2–5 of radiotherapy were significantly associated with OS.

**Table 2:** Univariable analysis of clinical, dosimetric and CBC variables with outcomes.

Variables	OS		LRFS		DMFS		PFS	
	Adj.*P*	HR (95% CI)	Adj.*P*	HR (95% CI)	Adj.*P*	HR (95% CI)	Adj.*P*	HR (95% CI)
Gender	0.572	0.857 (0.595-1.235)	0.968	1.010 (0.675-1.506)	0.125	0.709 (0.488-1.031)	0.727	0.891 (0.612-1.300)
Age (year)	0.777	0.998 (0.985-1.011)	0.890	0.998 (0.985-1.011)	0.058	0.984 (0.970-0.998)	0.312	0.991 (0.978-1.000)
ECOG PS	0.444	1.284 (0.800-2.060)	0.773	1.110 (0.710-1.747)	0.637	1.182 (0.732-1.907)	0.979	1.010 (0.654-1.55)
Family history	0.777	1.086 (0.673-1.753)	0.968	0.989 (0.595-1.645)	0.656	1.168 (0.712-1.914)	0.979	0.986 (0.603-1.610)
Hypertension	0.777	0.935 (0.658-1.329)	0.690	0.889 (0.602-1.311)	0.724	0.926 (0.638-1.346)	0.704	0.883 (0.618-1.260)
Diabetes	0.438	1.299 (0.804-2.099)	0.568	1.230 (0.740-2.046)	0.633	1.197 (0.730-1.964)	0.833	1.091 (0.667-1.790)
GTV (cc)	**<0.001**	1.003 (1.002-1.004)	**<0.001**	1.003 (1.002-1.004)	**<0.001**	1.003 (1.002-1.004)	**<0.001**	1.003 (1.002-1.004)
CCI	0.853	1.011 (0.897-1.14)	0.968	1.005 (0.886-1.140)	0.246	0.912 (0.801-1.037)	0.833	0.976 (0.865-1.100)
Body weight loss	0.572	1.401 (0.623-3.155)	0.690	1.290 (0.573-2.905)	0.667	1.242 (0.552-2.799)	0.926	0.917 (0.408-2.06)
Smoking (pack year)	0.432	1.149 (0.898-1.469)	0.536	1.128 (0.870-1.463)	0.699	0.941 (0.727-1.219)	0.833	1.047 (0.819-1.340)
Histology	0.734	0.953 (0.786-1.155)	0.445	0.890 (0.724-1.093)	0.076	1.230 (1.010-1.499)	0.727	1.056 (0.870-1.280)
T stage	**0.004**	1.209 (1.076-1.358)	**0.015**	1.202 (1.063-1.359)	**0.041**	1.161 (1.025-1.313)	**0.006**	1.197 (1.065-1.350)
N stage	0.734	1.053 (0.865-1.282)	0.537	1.103 (0.888-1.371)	0.130	1.217 (0.977-1.518)	0.282	1.154 (0.938-1.420)
Stage	**0.007**	1.346 (1.106-1.639)	**0.015**	1.374 (1.108-1.704)	**0.002**	1.474 (1.193-1.820)	**0.001**	1.501 (1.219-1.850)
Treatment sequence	**0.022**	0.774 (0.635-0.943)	**0.021**	0.749 (0.605-0.928)	**0.021**	0.750 (0.604-0.931)	**0.007**	0.736 (0.602-0.901)
Chemotherapy regimen	0.874	1.020 (0.833-1.249)	0.959	1.020 (0.815-1.276)	0.411	0.894 (0.720-1.110)	0.899	0.975 (0.790-1.203)
No of Chemo fractions	0.661	0.982 (0.932-1.035)	0.968	0.998 (0.947-1.052)	0.773	1.008 (0.957-1.063)	0.932	0.996 (0.949-1.050)
RT dose (Gy)	**0.007**	0.946 (0.911-0.982)	**0.035**	0.951 (0.914-0.9899)	**0.008**	0.940 (0.904-0.978)	**0.006**	0.943 (0.908-0.979)
No of RT fractions	0.181	1.036 (0.993-1.081)	0.495	1.022 (0.979-1.067)	0.130	1.041 (0.995-1.089)	0.257	1.030 (0.989-1.070)
Radiation technique	**0.013**	0.700 (0.543-0.903)	**0.018**	0.689 (0.529-0.8979)	**0.013**	0.680 (0.520-0.889)	**0.004**	0.663 (0.515-0.852)
MLD (Gy)	**<0.001**	1.110 (1.057-1.165)	**0.018**	1.074 (1.022-1.129)	**0.001**	1.111 (1.051-1.166)	**0.003**	1.086 (1.035-1.140)
MHD (Gy)	**<0.001**	1.027 (1.014-1.040)	**0.018**	1.018 (1.005-1.032)	**0.008**	1.021 (1.007-1.034)	**0.006**	1.020 (1.007-1.030)
MBD (Gy)	**<0.001**	1.179 (1.111-1.252)	**<0.001**	1.161 (1.088-1.238)	**<0.001**	1.120 (1.121-1.275)	**<0.001**	1.194 (1.122-1.270)
EDRIC (Gy)	**<0.001**	1.201 (1.129-1.278)	**<0.001**	1.161 (1.087-1.239)	**<0.001**	1.194 (1.118-1.275)	**<0.001**	1.190 (1.117-1.270)
PreRT.NLR	**<0.001**	1.098 (1.051-1.147)	**0.018**	1.067 (1.018-1.118)	**0.012**	1.072 (1.022-1.124)	**0.010**	1.066 (1.020-1.110)
PreRT.PLR	**0.004**	1.002 (1.001-1.004)	0.123	1.002 (1.000-1.003)	0.058	1.002 (1.000-1.003)	0.096	1.002 (1.001-1.003)
Week 1 NLR	0.171	1.019 (0.997-1.042)	0.495	1.012 (0.988-1.037)	0.656	1.008 (0.982-1.035)	0.727	1.007 (0.983-1.030)
Week 1 PLR	0.711	1.000 (0.999-1.001)	0.968	1.000 (0.999-1.001)	0.996	1.000 (0.999-1.001)	0.932	1.000 (0.999-1.000)
Week 2 NLR	**<0.001**	1.055 (1.030-1.081)	**0.007**	1.046 (1.018-1.074)	**0.014**	1.038 (1.011-1.066)	**0.002**	1.048 (1.021-1.080)
Week 2 PLR	**<0.001**	1.002 (1.001-1.002)	**0.018**	1.001 (1.000-1.002)	**0.020**	1001 (1.000-1.002)	**0.008**	1.001 (1.000-1.002)
Week 3 NLR	**<0.001**	1.049 (1.028-1.070)	**0.008**	1.035 (1.013-1.057)	**0.002**	1.041 (1.018-1.065)	**<0.001**	1.0431.022-1.060)
Week 3 PLR	**<0.001**	1.002 (1.001-1.002)	**<0.001**	1.001 (1.001-1.002)	**<0.001**	1.002 (1.001-1.002)	**<0.001**	1.002 (1.001-1.002)
Week 4 NLR	**<0.001**	1.057 (1.034-1.080)	**0.008**	1.038 (1.014-1.062)	**0.001**	1.046 (1.022-1.071)	**<0.001**	1.051 (1.028-1.080)
Week 4 PLR	**<0.001**	1.002 (1.001-1.002)	**<0.001**	1.001 (1.001-1.002)	**<0.001**	1.002 (1.001-1.002)	**<0.001**	1.002 (1.001-1.002)
Week 5 NLR	**0.007**	1.018 (1.006-1.030)	0.306	1.009 (0.996-1.022)	**0.008**	1.022 (1.008-1.036)	**0.002**	1.023 (1.010-1.040)
Week 5 PLR	**0.003**	1.000 (1.000-1.001)	0.265	1.000 (0.999-1.001)	**0.002**	1.001 (1.000-1.001)	**0.004**	1.001 (1.000-1.001)
Post.NLR	0.777	1.006 (0.968-1.045)	0.568	1.02 (0.9784-1.053)	0.370	1.021 (0.984-1.058)	0.401	1.019 (0.985-1.050)
Post.PLR	0.661	1.000 (0.999-1.001)	0.186	1.001 (1.000-1.002)	0.370	1.001 (1.000-1.001)	0.230	1.001 (0.999-1.000)

Adj. *P*, *P* value adjusted by Benjamini–Hochberg false discovery rate correction; ECOG PS, eastern cooperative oncology group performance status; CCI, charlson comorbidity index; Chemo, chemotherapy; RT, radiotherapy; MLD, mean lung dose; MHD, mean heart dose; MBD, mean body dose; EDRIC, estimated dose of radiation to immune cells; NLR, neutrophil-to-lymphocyte ratio; PLR, platelet-to-lymphocyte ratio. Values with *P* < 0.05 are presented in bold font.

For LRFS, elevated EDRIC, larger GTV, advanced T and clinical stages, reduced radiotherapy dose, 3DCRT, and treatment sequence all yielded significant associations. Distinct hematological predictors were observed for LRFS compared with OS: baseline NLR and PLR, as well as those tested at radiotherapy weeks 2, 3 and 4, were independent significant predictors, whereas week-5 NLR and PLR showed no statistical significance.

Univariate analyses of DMFS identified T stage, clinical stage, GTV, radiotherapy dose, treatment sequence and EDRIC as significant prognostic factors. Baseline and week 2–5 serial NLR and PLR also correlated significantly with DMFS.

In univariate models for PFS, significant predictors included EDRIC, GTV, T stage, clinical stage, treatment sequence, radiotherapy dose and 3DCRT. Serial inflammatory markers (NLR, PLR) collected at baseline and radiotherapy weeks 2 to 5 were additionally significantly associated with PFS.

### Clinical characteristics, dosimetric factors, and CBC parameters in relation to prognosis: a multivariate analysis

Collinearity diagnostics were conducted prior to univariate Cox regression screening. T stage was excluded from candidate covariates due to strong correlation with clinical stage. Week 4 NLR and week 4 PLR were also removed, given substantial multicollinearity with week 3 NLR and week 3 PLR, respectively.

According to Spearman’s correlation analysis, strong correlations were observed among the dosimetric parameters MLD, MHD, MBD, and EDRIC (*r* = 0.72, 0.78, 0.90; all *P* < 0.001). To independently assess the prognostic impact of EDRIC, two multivariate Cox regression models were established in this study: Model 1 included MLD, MHD, and MBD but excluded EDRIC, whereas Model 2 included EDRIC while excluding the three aforementioned dosimetric parameters. The multivariate Cox models were adjusted for statistically significant clinical characteristics, dosimetric factors, and CBC parameters derived from univariate analysis. The results of the multivariate analysis are summarized in **Table 3**.

**Table 3:** Multivariate Cox regression analysis of clinical features, dosimetric parameters and CBC parameters in models with and without the inclusion of EDRIC.

a.				
Variables	OS with EDRIC		OS without EDRIC	
	HR (95% CI)	Adj. *P*	HR (95% CI)	Adj. *P*
GTV (cc)	1.001 (1.000-1.003)	0.148	1.002 (1.000-1.004)	0.192
Stage	1.153 (0.925-1.436)	0.206	1.161 (0.932-1.446)	0.324
Treatment sequence	1.184 (0.715-1.958)	0.512	1.073 (0.641-1.797)	0.845
RT dose (Gy)	0.964 (0.925-1.004)	0.076	0.966 (0.927-1.008)	0.286
Radiation technique	0.571 (0.308-1.060)	0.076	0.645 (0.344-1.210)	0.324
PreRT NLR	1.082 (0.999-1.171)	0.054	1.077 (0.994-1.167)	0.221
PreRT PLR	0.998 (0.996-1.001)	0.178	0.998 (0.996-1.001)	0.328
Week 2 NLR	1.031 (0.981-1.083)	0.233	1.031 (0.979-1.086)	0.352
Week 2 PLR	0.999 (0.998-1.001)	0.253	0.999 (0.998-1.001)	0.453
Week 3 NLR	0.956 (0.913-1.002)	0.058	0.955 (0.912-1.001)	0.221
Week 3 PLR	1.002 (1.001-1.003)	**<0.001**	1.002 (1.001-1.003)	**<0.001**
Week 5 NLR	1.000 (0.967-1.035)	0.978	0.997 (0.963-1.032)	0.846
Week 5 PLR	1.000 (0.999-1.001)	0.821	1.000 (0.999-1.001)	0.845
MLD (Gy)			1.073 (1.000-1.153)	0.221
MHD (Gy)			1.012 (0.997-1.028)	0.286
MBD (Gy)			0.968 (0.865-1.084)	0.704
EDRIC (Gy)	1.111 (1.028-1.202)	**0.008**		
b.				
Variables	LRFS with EDRIC		LRFS without EDRIC	
	HR (95% CI)	Adj. P	HR (95% CI)	Adj. P
GTV (cc)	1.001 (1.000-1.002)	0.237	1.001 (1.000-1.003)	0.362
Stage	1.120 (0.883-1.421)	0.428	1.120 (0.881-1.424)	0.579
Treatment sequence	0.942 (0.529-1.679)	0.840	0.917 (0.508-1.658)	0.775
RT dose (Gy)	0.964 (0.924-1.006)	0.222	0.963 (0.922-1.006)	0.362
Radiation technique	0.701 (0.355-1.384)	0.421	0.720 (0.359-1.443)	0.579
PreRT NLR	1.019 (0.952-1.091)	0.641	1.018 (0.951-1.091)	0.650
Week 2 NLR	1.040 (0.986-1.097)	0.237	1.040 (0.984-1.098)	0.362
Week 2 PLR	0.999 (0.997-1.000)	0.222	0.999 (0.997-1.000)	0.362
Week 3 NLR	0.946 (0.901-0.993)	0.132	0.946 (0.900-0.993)	0.169
Week 3 PLR	1.002 (1.001-1.003)	**<0.001**	1.002 (1.001-1.003)	**<0.001**
MLD (Gy)			1.025 (0.951-1.104)	0.626
MHD (Gy)			1.006 (0.990-1.023)	0.626
MBD (Gy)			1.040 (0.921-1.173)	0.626
EDRIC (Gy)	1.090 (1.004-1.184)	**0.041**		
c.				
Variables	DMFS with EDRIC		DMFS without EDRIC	
	HR (95% CI)	Adj. *P*	HR (95% CI)	Adj. *P*
GTV (cc)	1.001 (1.000-1.002)	0.275	1.001 (1.000-1.003)	0.249
Stage	1.279 (1.016-1.611)	0.082	1.267 (1.006-1.596)	0.172
Treatment sequence	1.295 (0.711-2.360)	0.470	1.183 (0.638-2.194)	0.601
RT dose (Gy)	0.961 (0.921-1.002)	0.121	0.959 (0.919-1.001)	0.174
Radiation technique	0.473 (0.233-0.958)	0.082	0.522 (0.253-1.076)	0.195
PreRT NLR	1.025 (0.956-1.099)	0.53	1.021 (0.951-1.096)	0.601
Week 2 NLR	1.032 (0.978-1.090)	0.365	1.031 (0.975-1.090)	0.480
Week 2 PLR	0.998 (0.997-1.000)	0.082	0.998 (0.997-1.000)	0.172
Week 3 NLR	0.948 (0.902-0.996)	0.082	0.949 (0.902-0.999)	0.172
Week 3 PLR	1.003 (1.001-1.004)	**<0.001**	1.003 (1.001-1.004)	**<0.001**
Week 5 NLR	0.990 (0.955-1.026)	0.569	0.989 (0.954-1.026)	0.601
Week 5 PLR	1.000(1.000-1.001)	0.395	1.000 (1.000-1.001)	0.534
MLD (Gy)			1.058 (0.982-1.140)	0.255
MHD (Gy)			1.006 (0.990-1.022)	0.601
MBD (Gy)			1.033 (0.916-1.164)	0.601
EDRIC (Gy)	1.118 (1.031-1.212)	**0.046**		
d.				
Variables	PFS with EDRIC		PFS without EDRIC	
	HR (95% CI)	Adj. *P*	HR (95% CI)	Adj. *P*
GTV (cc)	1.001 (1.000-1.003)	0.154	1.001 (1.000-1.003)	0.276
Stage	1.234 (0.982-1.551)	0.154	1.229 (0.976-1.546)	0.276
Treatment sequence	1.142 (0.652-2.000)	0.760	1.102 (0.622-1.955)	0.853
RT dose (Gy)	0.956 (0.918-0.995)	0.113	0.954 (0.916-0.994)	0.195
Radiation technique	0.536 (0.277-1.038)	0.154	0.557 (0.284-1.091)	0.276
PreRT NLR	1.001 (0.938-1.069)	0.965	1.000 (0.936-1.068)	0.996
Week 2 NLR	1.032 (0.978-1.089)	0.357	1.030 (0.975-1.088)	0.482
Week 2 PLR	0.999 (0.998-1.000)	0.218	0.999 (0.998-1.000)	0.364
Week 3 NLR	0.963 (0.919-1.010)	0.218	0.965 (0.920-1.013)	0.364
Week 3 PLR	1.002 (1.001-1.003)	**<0.001**	1.002 (1.001-1.003)	**<0.001**
Week 5 NLR	1.009 (0.975-1.045)	0.760	1.008 (0.973-1.044)	0.818
Week 5 PLR	1.000 (0.999-1.001)	0.965	1.000 (0.999-1.001)	0.996
MLD (Gy)			1.031 (0.962-1.105)	0.573
MHD (Gy)			1.006 (0.991-1.022)	0.600
MBD (Gy)			1.063 (0.949-1.191)	0.482
EDRIC (Gy)	1.113 (1.03-1.202)	**0.045**		

Adj. *P*, *P* value adjusted by Benjamini–Hochberg false discovery rate correction; MLDm mean lung dose; MHD, mean heart dose; MBD, mean body dose; EDRIC, estimated dose of radiation to immune cells; NLR, neutrophil-to-lymphocyte ratio; PLR, platelet-to-lymphocyte ratio. Values with *P* < 0.05 are presented in bold font.

In both multivariate Cox models, only week 3 PLR and EDRIC remained independent prognostic factors for OS (**Table 3a**). In the multivariate model without EDRIC, week 3 PLR was significantly associated with OS, while MLD, MHD and MBD failed to achieve statistical significance. When EDRIC was incorporated into the regression model, EDRIC turned out to be another robust independent predictor alongside week 3 PLR.

Multivariate analyses for LRFS yielded consistent findings with the OS models (**Table 3b**), and EDRIC was confirmed to be an independent prognostic factor for LRFS. In the model without EDRIC, only week 3 PLR reached statistical significance, while MLD, MHD and MBD showed no predictive value. Moreover, week 3 PLR remained a robust independent predictor across both LRFS regression models.

In the DMFS multivariate model excluding EDRIC, only week 3 PLR was identified as significant variables, whereas MLD, MHD, and MBD were not. When EDRIC was included, it emerged as a significant factor, while week 3 PLR retained its significance.

Similarly, in the PFS multivariate analysis, EDRIC demonstrated strong predictive value. In the model including EDRIC, it served as a significant predictor alongside week 3 PLR. Conversely, in the model where EDRIC was replaced by MLD, MHD, and MBD, these dose parameters remained non-significant, whereas week 3 PLR continued to show significance.

In summary, this study consistently identified EDRIC as a key independent prognostic factor across four prognostic models. In contrast, traditional dosimetric parameters (MLD, MHD, and MBD) did not demonstrate independent predictive value in the LRFS, DMFS, and PFS models. Additionally, CBC parameters—particularly PLR at the third week of RT—showed significant prognostic value in the OS, LRFS, DMFS, and PFS models.

### Risk stratification of EDRIC and week 3 PLR for clinical prognostic evaluation

**Figure 1:**
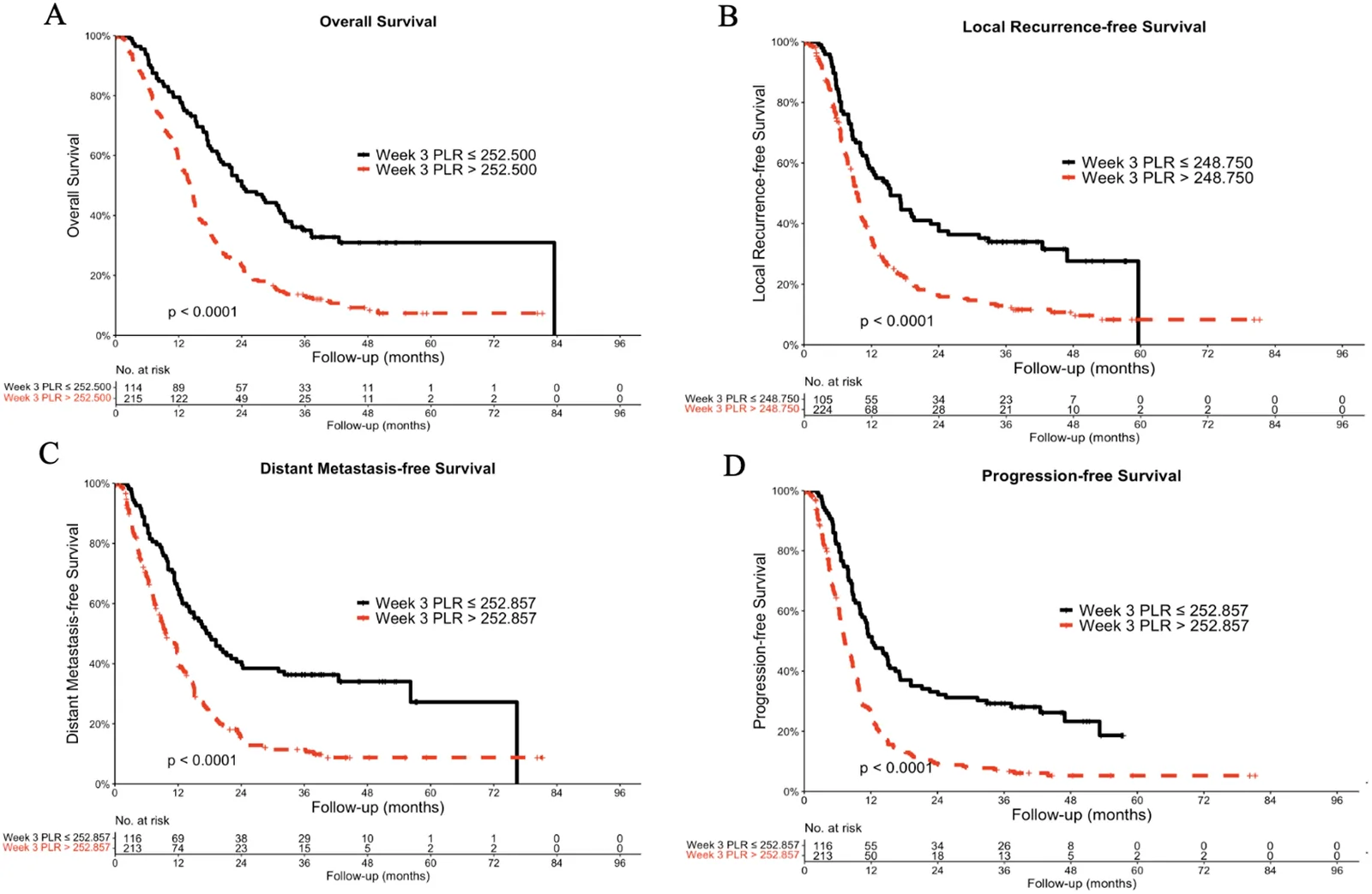
Kaplan–Meier survival curves for OS **(A)**, LRFS **(B)**, DMFS **(C)**, and PFS **(D)**, stratified by optimal cutoffs of week 3 PLR derived from maximally selected rank statistics.

**Figure 2:**
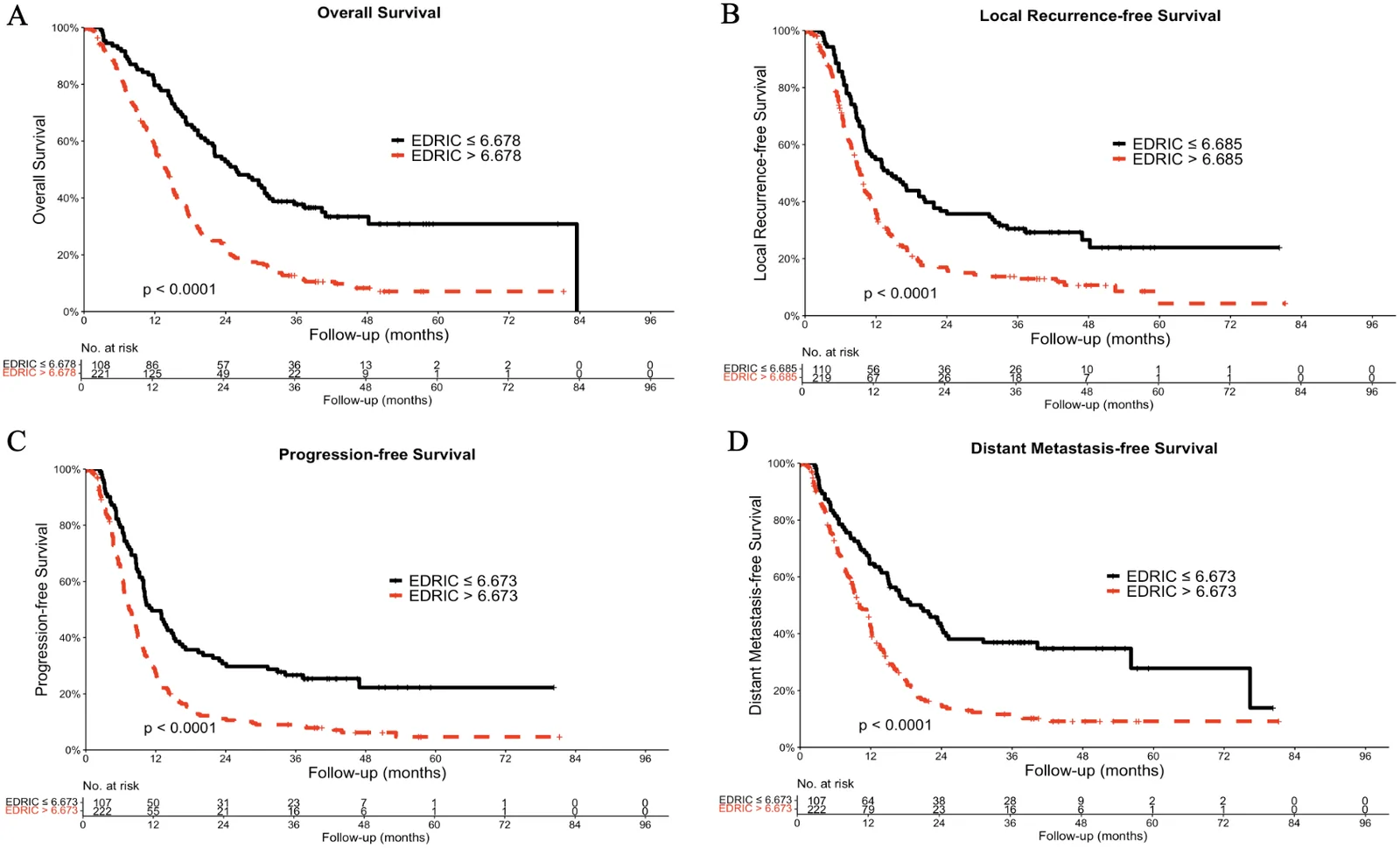
Kaplan–Meier survival curves for OS **(A)**, LRFS **(B)**, DMFS **(C)**, and PFS **(D)**, stratified by optimal cutoffs of EDRIC derived from maximally selected rank statistics.

Maximally selected rank statistics were applied to identify optimal dichotomization cutoffs for EDRIC and week 3 PLR across four clinical endpoints (OS, PFS, LRFS, and DMFS), and all derived thresholds are summarized in **Table 4**. Kaplan–Meier analyses stratified by these cutoffs showed that high-risk subgroups exhibited markedly poorer survival for every endpoint (all log-rank *P* < 0.001; **Figures 1**, **2**).

**Table 4:** Optimal cut-off values of EDRIC and Week 3 PLR across four survival endpoints.

Biomarker	Endpoint	Optimal cutoff	Low-risk n	High-risk n	Log-rank χ²	*P*
EDRIC	OS	6.678	108	221	36.85	**<0.001**
	LRFS	6.685	110	219	16.94	**<0.001**
	DMFS	6.673	107	222	26.49	**<0.001**
	PFS	6.673	107	222	23.11	**<0.001**
Week 3 PLR	OS	252.500	114	215	31.65	**<0.001**
	LRFS	248.750	105	224	19.02	**<0.001**
	DMFS	252.857	116	213	27.44	**<0.001**
	PFS	252.857	116	213	32.92	**<0.001**

Values with *P* < 0.05 are presented in bold font.

### C-index and calibration performance of the multivariable cox model

Fourteen candidate predictors covering clinical, dosimetric and hematological indicators were included for prognostic screening. Variables with *P* < 0.05 in univariate Cox regression were retained, and highly collinear variables with Spearman correlation coefficients > 0.7 were further excluded, leaving GTV, clinical stage, treatment sequence, RT dose, radiotherapy technique, serial on-treatment NLR/PLR, and EDRIC as core predictors. The C-index with corresponding 95% C-index confidence intervals for each multivariate model are summarized in **Table 5**.

**Table 5:** The prediction accuracy of significant clinical factors and combined model for OS, LRFS, DMFS and PFS prediction.

EDRIC	OS	LRFS	DMFS	PFS
C-index	0.622	0.604	0.617	0.620
95%CI	0.582-0.649	0.566-0.632	0.564-0.650	0.581-0.648
Combined model without EDRIC	OS	LRFS	DMFS	PFS
C-index	0.734	0.726	0.750	0.743
95%CI	0.700-0.755	0.707-0.752	0.726-0.764	0.720-0.760
Combined EDIRC model	OS	LRFS	DMFS	PFS
C-index	0.740	0.732	0.757	0.752
95%CI	0.712-0.758	0.714-0.754	0.739-0.773	0.735-0.768

Comparative analysis demonstrated that across all four survival endpoints, the model incorporating EDRIC yielded a higher C-index, suggesting that the addition of EDRIC improved the discriminative ability of the Cox proportional hazards regression model for patient survival outcomes. This improvement was particularly prominent for PFS, where the predictive benefit of EDRIC was most evident, elevating the C-index from 0.743 to 0.752. For OS, the predictive performance was also enhanced, with the C-index increasing from 0.734 to 0.740.

**Figure 3:**
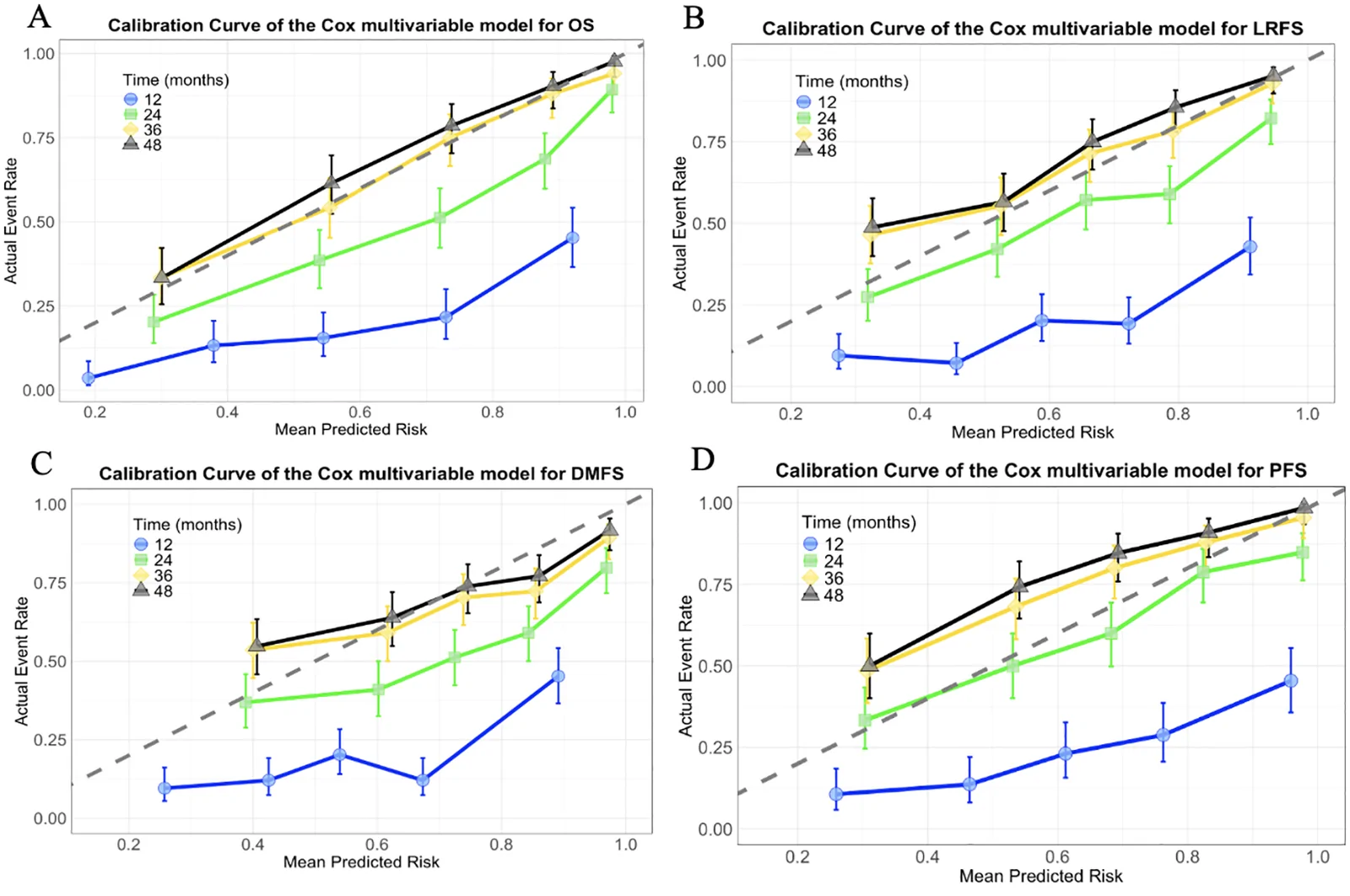
Calibration curves of the multivariate Cox regression model for predicting OS, LRFS, DMFS and PFS at different time points. **(A)** OS; **(B)** LRFS; **(C)** DMFS; **(D)** PFS.

Calibration curves with 95% Wilson confidence intervals for OS, LRFS, DMFS, and PFS demonstrated that the predicted risks derived from the multivariate Cox model rose in a time-dependent fashion (at 12, 24, 36, and 48 months) and gradually converged with the actual observed event rates within each risk stratum (**Figure 3**). Hosmer–Lemeshow goodness-of-fit tests yielded *P* values > 0.05 across all endpoints and all follow-up time points, indicating no statistically significant discrepancy between model-predicted probabilities and real observed event risks. At the 48-month landmark, predicted risks closely aligned with the actual incidence rates for all four clinical endpoints (OS: χ² = 0.079, *P* = 0.994; LRFS: χ² = 0.471, *P* = 0.925; DMFS: χ² = 0.356, *P* = 0.949; PFS: χ² = 1.156, *P* = 0.763), which further confirmed the favorable overall calibration performance of the constructed Cox models.

## Discussion

With the advent of the immunotherapy era, the immunomodulatory effects of RT have garnered widespread research attention. RT acts as a double-edged sword for antitumor immunity (21): it can trigger immunogenic cell death to activate systemic anti-tumor responses, yet excessive radiation to circulating immune cells also causes severe lymphopenia and immunosuppression. Traditional thoracic dosimetric parameters only evaluate normal lung parenchymal toxicity and cannot directly reflect radiation-induced immune injury, whereas EDRIC was developed to quantify radiation burden on immune system. Against this research backdrop, this study systematically evaluated the prognostic impacts of EDRIC, traditional dosimetric metrics, and serial systemic inflammatory markers via multivariate Cox regression modeling. The key results are summarized as follows: EDRIC appeared to be an independent prognostic factor for all four survival endpoints (OS, LRFS, DMFS, and PFS). By contrast, traditional dosimetric parameters (MLD, MHD, MBD) lost independent predictive significance after multivariate adjustment, as they cannot quantify radiation-induced injury to circulating immune cells, whereas systemic immunosuppression constitutes a critical pathway influencing long-term survival (22). Furthermore, among CBC-derived systemic inflammatory indices, week 3 PLR exhibited consistent prognostic significance, highlighting the potential role of dynamically monitored systemic inflammatory responses in tumor progression. Collectively, combining EDRIC with dynamically measured on-treatment inflammatory markers may help develop a more accurate prognostic stratification system for patients with LA-NSCLC undergoing thoracic chemoradiotherapy.

These observations raise a potential mechanistic interpretation. Conventional thoracic dosimetric parameters including MLD, MHD and MBD showed significant prognostic associations in univariate Cox analyses but failed to retain independent statistical significance after multivariate adjustment. Their prognostic effects are unlikely to stem solely from cardiopulmonary organ toxicity; instead, these parameters may indirectly reflect radiation exposure to immune system. As a metric dedicated to estimating radiation burden on circulating immune cells, EDRIC appeared to capture this immune-related prognostic signal. Additionally, incorporation of EDRIC improved model discrimination, especially for PFS and DMFS, and further gains were seen when EDRIC was combined with serial systemic inflammatory markers within our cohort.

In the current study, week 3 PLR, together with EDRIC, appeared to be an independent prognostic biomarker across all survival endpoints. Multiple prior investigations have validated that longitudinal fluctuations in NLR predict PFS and immune-related adverse events in radioimmunotherapy cohorts (23, 24). Consistent with these reports, univariate screening in our cohort identified serial on-treatment NLR as significantly correlated with prognosis; nevertheless, after multivariate adjustment, only week 3 PLR retained stable statistical significance. This discrepancy can be well explained by the distinct dynamic trajectories and biological characteristics of NLR and PLR throughout chemoradiotherapy. Thoracic chemoradiotherapy induces progressive, dose-dependent depletion of circulating lymphocytes, and absolute lymphocyte counts (ALC) may reach the nadir at approximately week 3 (25), representing the peak of systemic radiation-driven immunosuppression. During this critical treatment window, NLR is jointly shaped by dynamic changes in both neutrophils and lymphocytes following chemoradiotherapy. Given the biological complexity and functional plasticity of tumor-infiltrating neutrophils following radiation (26), multiple concurrent inflammatory perturbations may interfere with its prognostic performance. Such complexity renders week 3 NLR more susceptible to confounding factors, including baseline tumor burden and treatment-related systemic inflammation, which may obscure its predictive capacity in multivariable-adjusted models in our cohort.

In stark contrast, PLR predominantly reflects a persistently adverse inflammatory phenotype driven by radiation-mediated vascular endothelial injury, continuous lymphocyte depletion, and platelet-derived metastasis-promoting inflammatory cascades (27). Unlike NLR, PLR is not subject to functionally opposing signals from neutrophils that may offset its prognostic impact; its predictive signal therefore remains relatively stable and resistant to statistical interference in multivariable-adjusted models. The third week coincides with maximal treatment-related lymphopenia, fully capturing the peak of radiation-induced immune dysfunction that closely correlates with long-term tumor recurrence and distant metastasis, which explains why week 3 PLR maintained consistent unfavorable prognostic value across univariate tests, multivariate Cox models, and BH-adjusted analyses. Our findings are supported by two previous retrospective studies. One investigated NSCLC patients treated with ICIs plus opioids (28), while the other, by Yu et al., analyzed a large cohort of patients with limited-stage SCLC receiving definitive chemoradiotherapy (29). Both groups reported that PLR, but not NLR, retained independent prognostic significance in multivariate analyses. These consistent results reinforce the prognostic potential of PLR across different lung cancer subtypes and treatment modalities. Notably, both prior analyses focused on pretreatment inflammatory indices, whereas the present study evaluated dynamic week 3 PLR acquired during thoracic chemoradiotherapy.

Clinically, week 3 PLR bears multi-dimensional application value for routine toxicity surveillance and standardized risk stratification. First, it enables risk-stratified monitoring of chemoradiotherapy-related inflammatory toxicity. We established an optimal cutoff value for week 3 PLR specifically for patients receiving standard chemoradiotherapy, allowing clinicians to rapidly separate high-risk and low-risk inflammatory subgroups. Second, this marker guides the adjustment of chemotherapy intensity and treatment schedule. Patients with elevated week 3 PLR require close monitoring of systemic immune suppression, and their subsequent chemotherapy cycles can be delayed to avoid superimposed hematological toxicity; patients with low week 3 PLR can complete scheduled full-dose chemotherapy without interruption. Third, week 3 PLR can be delivered to mid-treatment MDT meetings as a routine auxiliary biomarker to tailor supportive care regimens and adjust long-term follow-up frequency based on individual risk levels. Sensitivity analyses and RCS regression validated single-cutoff grouping, rendering EDRIC and week 3 PLR feasible two-stage tools for adaptive personalized thoracic chemoradiotherapy.

EDRIC quantifies radiation-induced damage to the immune system and can be incorporated as a dedicated immune-sparing constraint during radiotherapy planning. In routine plan optimization, radiation physicists can prioritize minimizing EDRIC while maintaining adequate target coverage via standardized beam arrangement tuning, such as rational selection of beam angles, beam energy, and collimation parameters. This straightforward adjustment restricts unnecessary radiation exposure to circulating immune cells and lymphoid tissues from the outset of treatment, alleviating RT-triggered systemic immunosuppression.

Crucially, week 3 PLR can serve as dynamic real-time feedback to trigger adaptive radiotherapy re-planning. A marked rise in week 3 PLR implies severe treatment-induced immune dysregulation, which usually coincides with excessive radiation burden to immune compartments (high EDRIC). For such high-risk patients, clinicians can perform adaptive plan revisions to further cut EDRIC, moderately reduce prescription dose, or shrink target margins to alleviate immune injury. For patients with low week 3 PLR, reflecting mild immune suppression, full-dose radiotherapy can be preserved, and combination immunotherapy can be safely administered to amplify anti-tumor immunity without extra immune-sparing dose modifications. Advanced delivery modalities such as proton therapy (30, 31) and spatially fractionated lattice radiotherapy (32, 33) may further optimize EDRIC reduction and synergize with immunotherapy, yet their routine clinical application remains to be validated in larger prospective cohorts. Taken together, EDRIC offers static immune dosimetric references for radiotherapy planning, whereas week 3 PLR provides dynamic intra-treatment immune signals. The two markers may work in tandem to enable stratified, individualized immune-protective radioimmune therapy, which could yield clinical benefits even if each biomarker alone shows only modest hazard ratios.

Several limitations of the present study warrant acknowledgment, despite its novel findings regarding immune dosimetry and dynamic inflammatory markers for LA-NSCLC risk stratification. First, given the retrospective single-center design, our results are potentially prone to selection bias. In addition, incomplete serial complete blood count sampling led to reduced sample sizes at later follow-up time points, which may further introduce subtle selection bias and affect the analytical robustness of dynamic inflammatory biomarker analyses. Second, key molecular covariates, including EGFR mutation status and PD-L1 expression, were unavailable for multivariate analysis due to data limitations, which may restrict the generalizability of our findings. Third, incomplete documentation of competing mortality events precluded competing risk regression analyses for individualized prediction of non-OS endpoints. Fourth, only internal validation was performed, and external independent cohorts were lacking to further verify the predictive robustness of EDRIC and week 3 PLR. Fifth, potential interactions between chemotherapy subtypes and EDRIC require further investigation in larger cohorts. Finally, the current EDRIC framework remains relatively simplified. As a single aggregate dosimetric parameter, it does not fully account for radiation exposure to immune-related subcompartments, including lymph nodes, great vessels, spleen, and red bone marrow, nor does it incorporate tumor-infiltrating lymphocytes within the tumor microenvironment (34). Future work may integrate HEDOS, a computational tool quantifying radiation exposure to circulating blood cells (35), to refine blood dose estimation and build more comprehensive dosimetric immune models. Prospective multicenter studies with external validation in radioimmunotherapy cohorts will further strengthen the reliability and generalizability of our combined immune-dosimetric and inflammatory biomarker model.

## Conclusions

This study suggests that EDRIC may serve as an independent prognostic biomarker associated with OS, LRFS, DMFS and PFS in patients with LA-NSCLC treated with definitive thoracic chemoradiotherapy. A prognostic model integrating EDRIC with on-treatment inflammatory markers (NLR and PLR) demonstrates improved predictive performance compared with EDRIC alone. This combined framework may facilitate individualized risk stratification and offer preliminary evidence to support clinical decision-making. Large-scale prospective multicenter investigations, including external validation within radioimmunotherapy cohorts, are needed to refine this integrated model prior to widespread clinical implementation.

## Data Availability

The original contributions presented in the study are included in the article/**Supplementary Material**, further inquiries can be directed to the corresponding author.
