# Supplementary material for: Study on the effects of estimated dose of radiation to immune cells and CBC parameters on the prognosis of patients with locally advanced non-small cell lung cancer undergoing radiotherapy
Source: Front Immunol. 2026 Aug 19;17:1852238. doi: 10.3389/fimmu.2026.1852238 (PMC13534069; doi:10.3389/fimmu.2026.1852238)
Supplement: Supplementary file 1 [file Table1.docx]

Supplementary Table S1

Median and interquartile range (IQR) of serial neutrophil-to-lymphocyte ratio (NLR) and platelet-to-lymphocyte ratio (PLR) at predefined blood sampling time points

| Biomarker | Time point | Median (IQR) |
| --- | --- | --- |
| NLR | Pre-RT | 3.73(1.86-4.55) |
| PLR | Pre-RT | 197.20(108.00-198.81) |
| NLR | Week 1 during RT | 5.34(2.55-6.44) |
| PLR | Week 1 during RT | 287.50(134.64-282.50) |
| NLR | Week 2 during RT | 6.31(3.00-7.60) |
| PLR | Week 2 during RT | 314.40(182.71-366.00) |
| NLR | Week 3 during RT | 10.00(3.43-9.23) |
| PLR | Week 3 during RT | 443.33(216.56-469.17) |
| NLR | Week 4 during RT | 8.98(4.11-10.46) |
| PLR | Week 4 during RT | 343.75(224.38-454.58) |
| NLR | Week 5 during RT | 8.10(4.35-10.08) |
| PLR | Week 5 during RT | 506.00(221.56-513.33) |
| NLR | 1-month post-RT | 2.92(2.73-5.09) |
| PLR | 1-month post-RT | 200.83(153.33-292.50) |

Abbreviations: IQR, interquartile range; NLR, neutrophil-to-lymphocyte ratio; PLR, platelet-to-lymphocyte ratio; RT, radiotherapy.
